# Suicidal Mortality and Motives Among Middle-School, High-School, and University Students

**DOI:** 10.1001/jamanetworkopen.2023.28144

**Published:** 2023-08-07

**Authors:** Motohiro Okada, Ryusuke Matsumoto, Takashi Shiroyama, Eishi Motomura

**Affiliations:** 1 Division of Neuroscience, Department of Neuropsychiatry, Graduate School of Medicine, Mie University, Tsu, Japan

## Abstract

**Importance:**

The suicide mortality rate per 100 000 population (SMRP) consistently decreased before the COVID-19 pandemic outbreak in Japan and then unexpectedly increased during the pandemic. However, the underlying mechanisms remain poorly understood.

**Objective:**

To identify trends in and factors associated with suicidal mortality and motives among students in Japan from 2007 to 2022.

**Design, Setting, and Participants:**

In this cross-sectional study, data on SMRPs among Japanese middle-school, high-school, and university students were obtained from the government suicide database Suicide Statistics of the National Police Agency.

**Main Outcomes and Measures:**

Age-dependent and temporal fluctuations in annual SMRPs, disaggregated by suicidal motive (7 categories and 52 subcategories), sex, and school, were analyzed using linear mixed-effect and joinpoint regression models, respectively.

**Results:**

Total suicide numbers from 2007 to 2022 were as follows: 760 male middle-school students, 635 female middle-school students, 2376 male high-school students, 1566 female high-school students, 5179 male university students, and 1880 female university students. The mean (SD) student populations from 2007 to 2022 were as follows: 1 752 737 (81 334) male middle-school students, 1 675 572 (78 824) female middle-school students, 1 648 274 (67 520) male high-school students, 1 614 828 (60 032) female high-school students, 1 652 689 (32 724) male university students, and 1 229 142 (57 484) female university students. Among male students, the leading motives were school-related factors (underachievement and worrying about the future), followed by family-related and health-related motives. Among female students, school-related and family-related motives decreased, but health-related motives showed an age-dependent increase. The SMRPs of middle-school male students and female students were almost equal (mean [SD], 2.7 [1.0] vs 2.4 [1.4]), but the age-dependent increase in SMRPs among male students was pronounced (mean [SD], high-school vs university male students, 9.1 [2.4] vs 19.6 [3.0]; high-school vs university female students, 6.1 [2.4] vs 9.6 [1.8]). However, the incidence of suicide among high-school students associated with health-related motives was greater in female students. The majority of suicides associated with major impactable suicidal motives (school-related, health-related, and family-related motives) began increasing before the pandemic. Changes in SMRP associated with interpersonal relationships, such as conflict with classmates or parents, were not significant, but the rates increased greatly during the pandemic.

**Conclusions and Relevance:**

School-related, health-related, and family-related problems were major motives, whereas the impacts of health-related and family-related motives increased and decreased with age, respectively. Notably, most SMRPs associated with major impactable motives (underachievement, conflict with a parent or classmate, and mental illnesses) had already begun increasing in the late 2010s, indicating that recent increasing SMRPs among school-aged individuals were associated with pandemic-related factors and other factors affecting this generation before the pandemic. It may be inappropriate to uniformly apply research findings based on school-aged individuals to school-based suicide prevention programs for students in middle school, high school, and university.

## Key Points

**Question:**

What factors are associated with the increasing number of suicides among individuals younger than 30 years in Japan during the COVID-19 pandemic?

**Findings:**

In this cross-sectional study of 12 396 middle-school, high-school, and university students, from 2007 to 2022, suicide mortality rates have consistently increased since the late 2010s, with major associated factors including school-related, health-related, and family-related problems; however, the factors associated with suicide among students change according to their life stage (ie, life cycle).

**Meaning:**

These findings suggest that designing and implementing school-based suicide prevention programs that are effective for the specific vulnerabilities in psychological and social developmental stages of middle-school, high-school, and university students can contribute to suicide prevention for students.

## Teaser

This cross-sectional study examines trends in and factors associated with suicidal mortality and motives among middle-school, high-school, and university students in Japan, from 2007 to 2022.

## Introduction

From 2009 to 2019, Japan’s governmental comprehensive regional suicide prevention programs^1,2,3,4,5,6,7,8,9^ were associated with an approximately 30% decrease in the suicide mortality rate per 100 000 population (SMRP). The main frameworks of suicide prevention programs were enhancing regional (prefectural and municipal) welfare and social safety nets and protection systems, including personal and telephone and internet consultations, development of gatekeepers, enlightenment, and specific interventions.^3,4,5^ However, the SMRP increased after the COVID-19 pandemic outbreak.^1,2,10,11,12,13,14^

During the initial stage of the pandemic (2020-2021), the SMRP of working-age male individuals continuously decreased, but SMRPs for working-age female individuals and individuals younger than 30 years increased.^1,2,10,11,12,13,14^ The SMRP was 2.5 times greater among male individuals vs female individuals during 2009 to 2019,^4,6^ but the differential decreased to 2.0 in 2021,^10,12,13^ indicating that the increase in SMRP during the pandemic was more pronounced among female individuals. Indeed, before the pandemic (2016-2019), the SMRPs were 2.5 among individuals younger than 20 years and 3.8 among those aged 20 to 29 years; during the pandemic (2020-2022), SMRPs were 17.2 among those younger than 20 years and 21.3 among those aged 20 to 29 years.^1,2,10,11,12,13,14^ Therefore, the young generation (ie, individuals aged <30 years) is currently considered a high-risk group.^10,11,12,13,14,15^ With the SMRPs of the young generation increasing, both the Ministry of Education, Culture, Sports, Science and Technology and Ministry of Health, Labor and Welfare enhanced school mental health supporting systems and developed crisis line resources using the internet^16,17^; however, the increasing trends in SMRPs in the young generation remain unsuppressed.^10^ Thus, governmental suicide prevention programs could not decrease SMRPs in the young generation.^4,5,18^ In addition, the mechanisms underlying the increased SMRPs and reasons why suicide prevention programs could not decrease SMRPs in the young generation should be elucidated.

Valid analyses of direct causality for increasing SMRPs in Japan are needed for evidence-based implementation of suicide prevention programs.^10,11,12,19,20,21^ Schools have been considered to be the most effective organizations for modifying environmental and psychological factors associated with suicides via standardized suicide prevention programs.^19,22,23^ The underlying mechanisms in adolescent suicides are complicated owing to their complex contexts, including specific physical, psychological, social, and educational developmental statuses.^24^ Therefore, it might be inappropriate to uniformly apply findings obtained from research on adolescents to suicide prevention programs for middle-school, high-school, and university students. We investigated temporal fluctuations in SMRPs disaggregated by suicidal motive, sex, and school, as published in the government suicide database, Suicide Statistics of the National Police Agency (SSNPA).^25^

## Methods

This cross-sectional study adhered to the Strengthening the Reporting of Observational Studies in Epidemiology (STROBE) reporting guideline. The medical ethics review committee of Mie University waived requirements for informed consent and ethical approval because these are publicly available governmental data.

Annual suicide numbers disaggregated by motive, sex, and school in Japan from 2007 to 2022 were obtained from the SSNPA.^25,26^ SSNPA provides annual suicide numbers in Japan disaggregated by various factors, including sex (male and female), school (middle school, high school, and university), and suicidal motives (7 categories: family, health, economic, employment, romance, school related, and other motives, with 52 subcategories) (eTables 1, 2, and 3 in Supplement 1).^8,16,18,25,26,27,28,29^ The detailed methods of the investigation of suicidal motives are explained in the eAppendix in Supplement 1. Annual student populations in middle school, high school, and university from 2007 to 2022 (denominator for SMRP derivation) were obtained from the School Basic Survey, a government database of the Ministry of Education, Culture, Sports, Science and Technology.^30^ SMRP was calculated by dividing the annual suicide numbers of the target group by the annual populations of the same target group in the same year (eg, the annual suicide number among male university students in 2020 was divided by the total number of male university students in 2020). SSNPA released annual suicide numbers for which motives were determined.

### Statistical Analysis

In this study, the trends, discontinuity, and their effect size of SMRPs of male and female middle-school, high-school, and university students from 2007 to 2022 were analyzed by joinpoint regression analysis (JPRA) using the Joinpoint Regression Program version 5.0.2 (National Cancer Institute).^10,12,31,32^ The differences in SMRPs among sexes (male and female students) and schools (middle school, high school, and university) from 2007 to 2022 were compared using linear mixed-effect models with the Scheffe post hoc test using SPSS statistical software for Windows version 27 (IBM).^11,13^ The detailed statistical methods are described in the eAppendix in Supplement 1. Two-tailed *P* < .05 was considered statistically significant.

## Results

### Fluctuations in SMRPs Disaggregated by Sex and School

**Figure 1.**
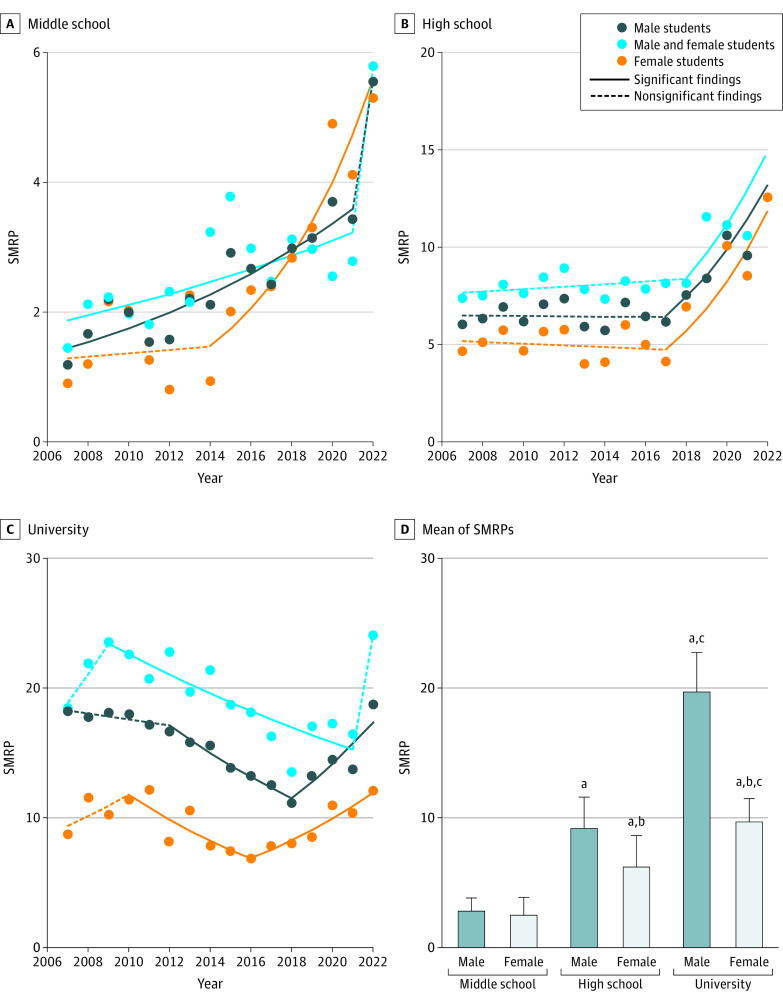
Mean and Fluctuations of Suicide Mortality Rate per 100 000 Population (SMRP) of Japanese Students, 2007-2022 Graphs show temporal fluctuations of SMRPs of middle-school (A), high-school (B), and university (C) students and mean of SMRPs (D) from 2007 to 2022 in Japan. Statistical significance was set at *P* < .05 for trends of SMRPs detected by joinpoint regression analysis. In panel D, *P* values were calculated using linear mixed-effect model with Scheffe post hoc test. ^a^*P* < .05 vs middle-school students of same sex. ^b^*P* < .05 vs male students of same school group. ^c^*P* < .05 vs high-school students of same sex.

Total suicides from 2007 and 2022 reported in SSNPA were as follows: 760 male middle-school students, 635 female middle-school students, 2376 male high-school students, 1566 female high-school students, 5179 male university students, and 1880 female university students (eTables 1, 2, and 3 in Supplement 1). The mean (SD) student populations from 2007 to 2022 were as follows: 1 752 737 (81 334) male middle-school students, 1 675 572 (78 824) female middle-school students, 1 648 274 (67 520) male high-school students, 1 614 828 (60 032) female high-school students, 1 652 689 (32 724) male university students, and 1 229 142 (57 484) female university students. Considering that each student accounted for approximately 2% to 3% of the Japanese population (eTables 1, 2, and 3 in Supplement 1), the student SMRPs were smaller than the national-level SMRP. Student SMRPs of both sexes showed an age-dependent increase (Figure 1). SMRPs of middle-school students were almost equal between male and female students (mean [SD], 2.7 [1.0] vs 2.4 [1.4]) but the age-dependent increase in SMRPs among male students was pronounced, and male high-school and university students showed higher SMRPs than female students (mean [SD], high-school vs university male students, 9.1 [2.4] vs 19.6 [3.0]; high-school vs university female students, 6.1 [2.4] vs 9.6 [1.8]) (Figure 1 and eFigure 1A in Supplement 1).

SMRPs of middle-school students of both sexes consistently increased during 2007 to 2022, further increased in 2014 and 2021, and were detected as joinpoints by JPRA (Figure 1 and eTable 4 in Supplement 1). SMRPs of high-school students of both sexes turned from unchanging to increasing in 2017. The female university student SMRP turned from decreasing to increasing in 2016, whereas that of male students consistently decreased from 2009 to 2021 but sharply (although nonsignificantly) increased in 2022, because JPRA cannot analyze slope between only 2 periods (Figure 1).

Among male students, school-related factors were consistently the leading motive (372 middle-school students [49%], 1089 high-school students [46%], and 2193 university students [42%]) (eFigure 1A in Supplement 1). Among female middle-school students, school-related factors were also the leading motive (289 students [45%]). Impacts of school-related (493 students [32%]) and health-related (523 students [34%]) motives were almost equal in female high-school students, whereas health-related factors (725 students [39%]) were the leading motive among female university students, followed by school-related factors (eFigure 1A in Supplement 1). Impacts of other suicidal subcategories in school-related, health-related, and family-related motives on SMRPs are indicated in eFigure 1 in Supplement 1.

### Fluctuations in SMRPs Associated With School-Related Motives

Among school-related factors, underachievement was consistently the leading motive for suicide among male students, followed by worrying about the future and conflict with classmates (eFigure 1B in Supplement 1). Among female students, the rank-order of impacts of school-related motives transformed with age, shifting from conflict with classmates (77 students [26%]), underachievement (58 students [20%]), and worrying about the future (46 students [16%]) among middle-school students; to worrying about the future (126 students [25%]), conflict with classmates (103 students [21%]), and underachievement (92 students [19%]) among high-school students; and finally to worrying about the future (207 students [38%]), underachievement (174 students [32%]), and conflict with classmates (68 students [13%]) among university students (eFigure 1B in Supplement 1).

**Figure 2.**
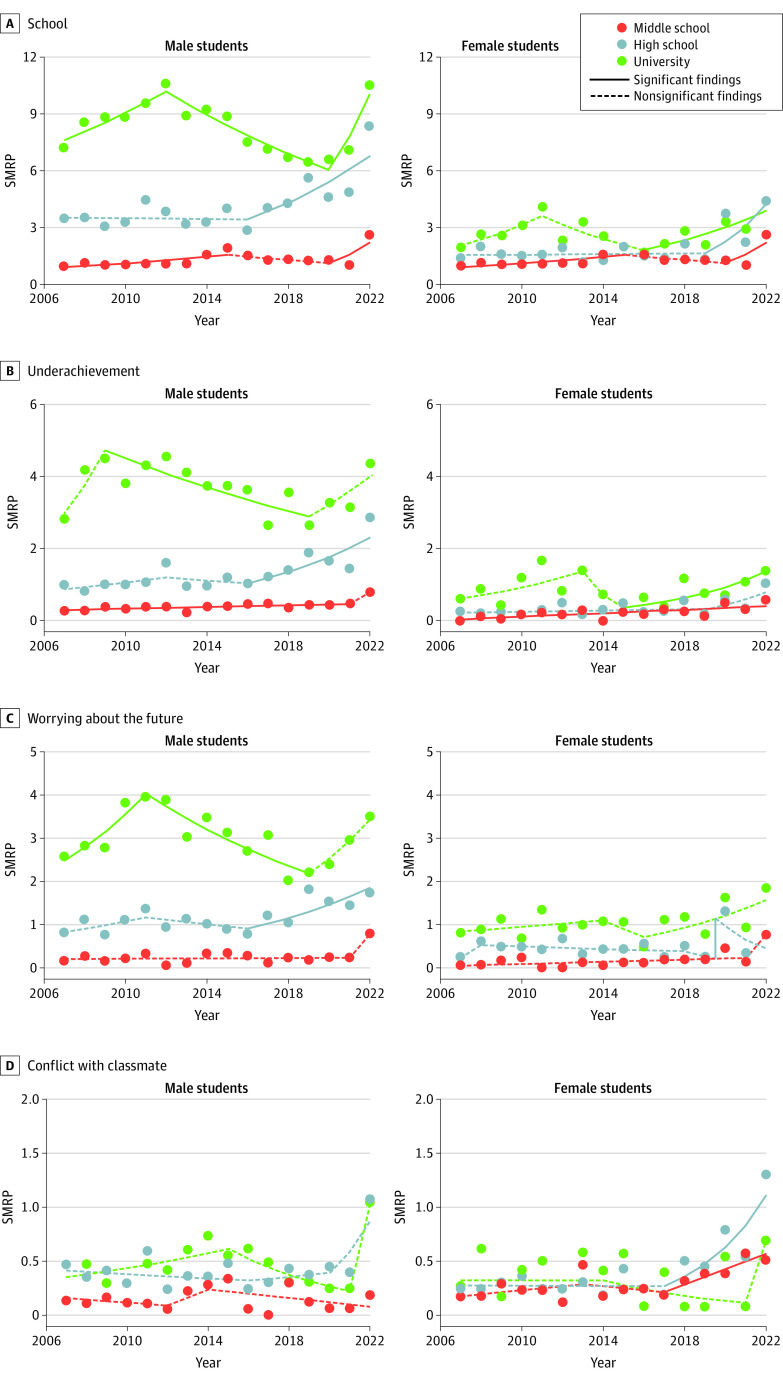
Fluctuations of Suicide Mortality Rate per 100 000 Population (SMRP) Associated With School-Related Factors Among Japanese Students, 2007-2022 Graphs show fluctuations of SMRPs associated with school-related factors (A), underachievement (B), worrying about the future (C), and conflict with classmates (D). Statistical significance was set at *P* < .05 for trends of SMRPs detected by joinpoint regression analysis.

SMRPs associated with school-related motives among female middle-school and university students began increasing in 2012 and 2016, respectively. Those of male students changed from decreasing to increasing in 2020 (Figure 2 and eTable 4 in Supplement 1). Those of male and female high-school students increased in 2016 and 2019, respectively.

Of note, SMRPs associated with conflict with classmate of female middle-school and high-school students were greater compared with male student in the late 2010s. Suicides among male and female university students associated with conflict with classmates increased greatly in 2022 (Figure 2).

### Fluctuations in SMRPs Associated With Health-Related Motives

**Figure 3.**
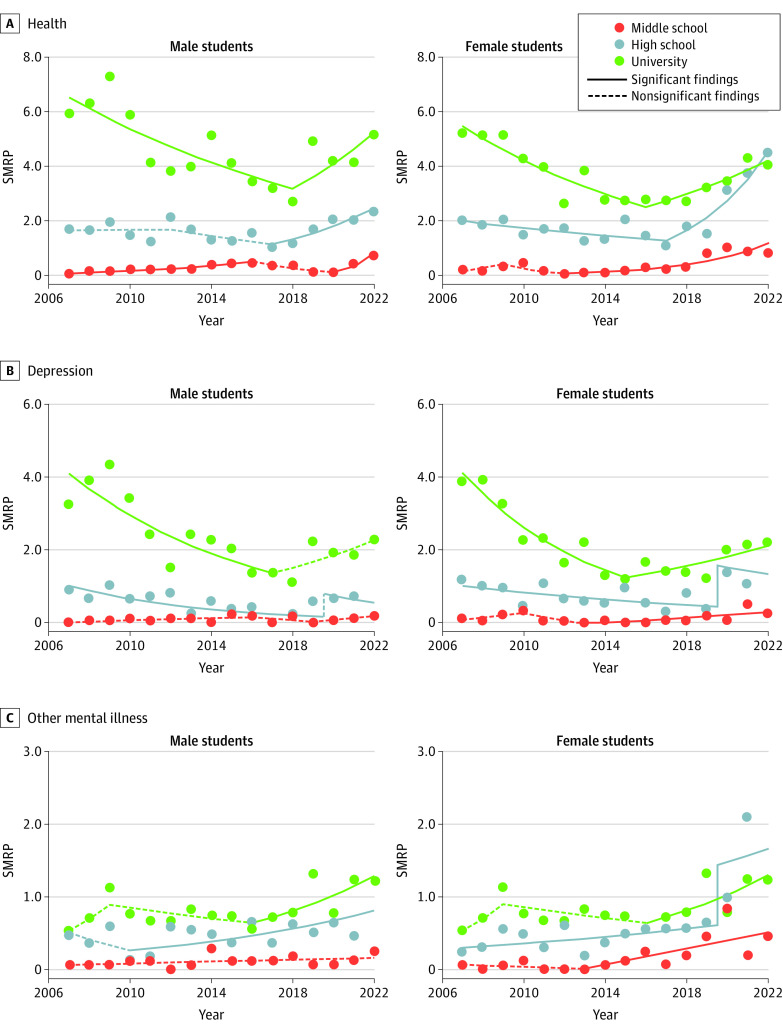
Fluctuations of Suicide Mortality Rate per 100 000 Population (SMRP) Associated With Health-Related Factors Among Japanese Students, 2007-2022 Graphs show fluctuations of SMRPs associated with health-related factors (A), depression (B), and other mental illness (C). Statistical significance was set at *P* < .05 for trends of SMRPs detected by joinpoint regression analysis.

Among health-related motives, depression and other mental illnesses were leading factors associated with student SMRPs. Their impacts increased in an age-dependent manner (eFigure 1C in Supplement 1). SMRPs of female high-school students associated with health-related motives, including depression and other mental illness, were markedly higher than in male students (Figure 3 and eFigure 1C and eFigure 2 in Supplement 1).

SMRPs associated with health-related factors among male and female middle-school students began increasing in 2020 and 2012, respectively (Figure 3; eTable 4 in Supplement 1). SMRPs associated with health-related factors among male and female high-school students turned from decreasing to increasing in 2017, whereas those of male and female university students turned from decreasing to increasing in 2018 and 2015, respectively (Figure 3). SMRPs associated with health-related factors, including depression and other mental illnesses, among female middle-school and high-school students were already larger than in male students in the late 2010s. SMRPs associated with depression and other mental illness among high-school female students displayed positive discontinuation synchronized with the COVID-19 outbreak (Figure 3).

### Fluctuations in SMRPs Associated With Family-Related Factors

Among family-related factors, except for male middle-school students, conflict with parents was the leading factor associated with student SMRPs (153 male high-school students [42%], 118 male university students [31%], 85 female middle-school students [50%], 113 female high-school students [42%], and 53 female university students [34%]), followed by severe verbal reprimand (95 male high-school students [26%], 83 male university students [22%], 47 female middle-school students [28%], 58 female high-school students [22%], and 19 female university students [12%]), and conflict with other family members (21 male middle-school students [10%], 35 male high-school students [10%], 43 male university students [11%], 17 female middle-school students [10%], 43 female high-school students [16%], and 30 female university students [19%]) (eFigure 1D in Supplement 1). Severe verbal reprimand was the leading factor in male middle-school students (105 students [49%]), followed by conflict with parents (62 students [29%]) and conflict with other family members (21 students [10%]) (eFigure 1D in Supplement 1). Remarkably, SMRPs associated with other suicide motives increased in an age-dependent manner, whereas the age-dependent increase in SMRPs associated with family-related motives was indistinct; rather, their impact decreased in an age-dependent manner.

**Figure 4.**
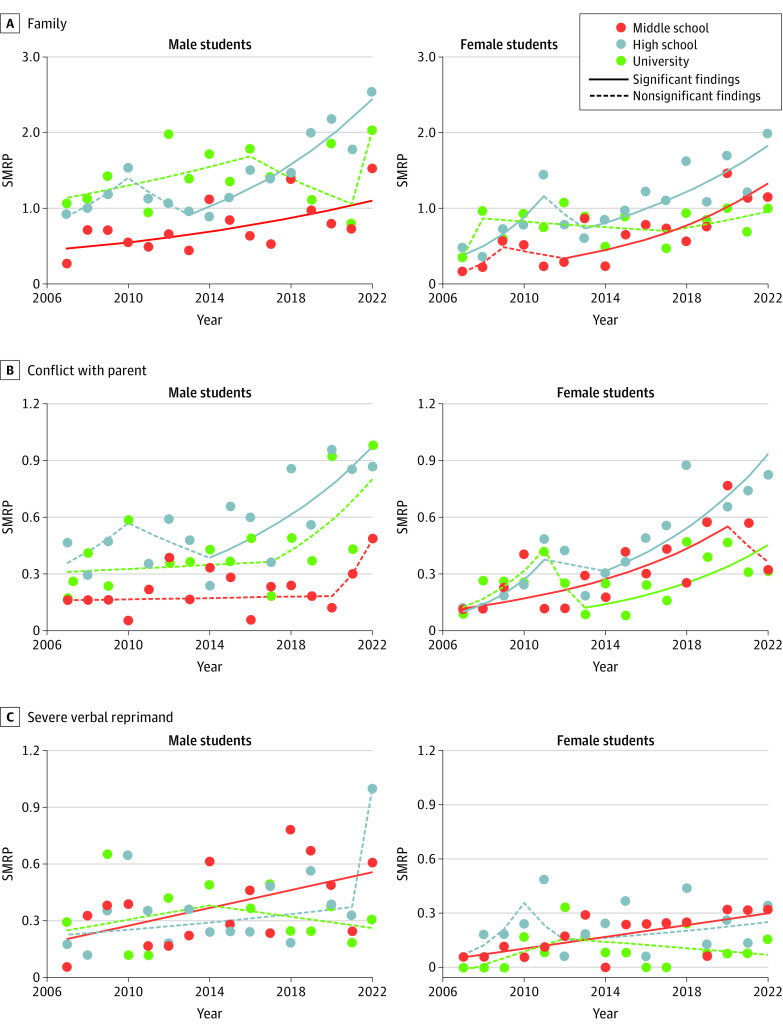
Fluctuations of Suicide Mortality Rate per 100 000 Population (SMRP) Associated With Family-Related Factors Among Japanese Students, 2007-2022 Graphs show fluctuations of SMRPs associated with family-related factors (A), conflict with parents (B), and severe verbal reprimand (C). Statistical significance was set at *P* < .05 for trends of SMRPs detected by joinpoint regression analysis.

SMRPs associated with family-related motives of middle-school and high-school students increased in the early 2010s, but those of university students did not significantly change (Figure 4 and eTable 4 in Supplement 1). SMRPs associated with conflict with parents in groups other than middle-school and university male students increased from the late 2010s, whereas those in middle-school and university male students showed large increases that were not statistically significant after the pandemic outbreak (Figure 4). SMRPs associated with severe verbal reprimand of middle-school students of both sexes consistently increased from 2007 to 2022. SMRPs of high-school and university students showed no significant change, but a sharp increase was observed in male high-school students in 2022 (Figure 4).

## Discussion

This cross-sectional study elucidated the sex-dependent and school-dependent specific features of SMRPs among students in Japan from 2007 to 2022. First, SMRPs increased with age from middle school to high school to university. SMRPs of male and female middle-school students were almost equal, but the age-dependent increase in SMRPs among male students was more pronounced than that among female students. Second, from 2007 to 2022, SMRPs among middle-school and high-school students of both sexes increased, but the majority of joinpoints of enhanced increasing trends (school-related, health-related, and family-related motives) were detected before the pandemic. SMRPs among female university students turned from decreasing to increasing in 2016; however, that of male students consistently decreased from 2009 to 2021, but then increased sharply in 2022. Similar increasing patterns (sharp increase) of SMRPs associated with subcategorized motives were predominantly observed among male students. These findings indicate a disproportionate increase in SMRPs among female students before the pandemic outbreak.^10,11,12,13^ Third, generally, SMRPs for male students were larger than those for female students; however, the SMRPs associated with health-related factors, including depression and other mental illness, for middle-school and high-school female students were greater than those for male students. Furthermore, the SMRP associated with conflict with parent among middle-school female students was larger than that among male students. Accordingly, mental health impairment of female middle-school and high-school students plays an important role in their increased SMRPs. Regarding the increased SMRPs around the pandemic in Japan, it should be emphasized that they are exceptional in the global context. Thus far, most studies from other Organisation for Economic and Co-operation and Development countries have reported that SMRPs at the national level and in the young generation were decreasing or remained unchanged during the pandemic.^33,34,35,36,37^

In the late 2010s, SMRPs associated with health-related factors, including depression and other mental illness, increased among female middle-school and high-school students compared with male students. Therefore, increasing internalization symptoms or disorders of female students may be an underlying factor associated with increasing SMRPs among female students. In the relevant literature,^38^ the age of onset of internalization disorders was approximately 15 years; there was no sex difference in their prevalence during elementary school. During high school, the prevalence of internalization disorders was significantly higher in female than in male students.^39,40^ Temporal fluctuation patterns between the prevalence of internalization disorders and increasing SMRPs associated with health-related factors are remarkably consistent. Furthermore, the Patient Survey of the Ministry of Health, Labor and Welfare reported that the prevalence of psychiatric disorders in female students aged 10 to 24 years in 2020 increased compared with that in 2017 (eFigure 2 in Supplement 1).^41^ Internalization symptoms are established suicidal risks.^42,43,44^ Anxiety plays important roles in the transition from suicidal ideation to suicidal behaviors^43^ and is a factor associated with lifetime risk of suicidal ideation and behaviors.^44^ Retrospective studies in which families were interviewed reported the presence of psychiatric disorders in 90% of individuals who died by suicide.^45^ Students perceive increasing their internalization symptoms at the transition from middle school to high school, with larger impacts on female than male students, who are focusing on future academic and career options with age.^46,47^ Therefore, the peculiar mentality of high-school students probably contributes to increasing SMRPs associated with health-related and school-related factors and the pronounced increase in SMRPs in female students in the late 2010s. In addition, the stress factors associated with the pandemic,^48^ including the psychological burden of the pandemic itself and poor adaptation to school closures or changing educational opportunities, might adversely affect the mental health and resilience of high-school female students, resulting in increases in their SMRPs associated with depression, other mental health issues, and worrying about the future.

However, suicides, including suicidal ideation and behavior, are rarely explained by a single factor, and most contemporary theories of suicide emphasize interactions among several biological, environmental, social, and/or psychological factors.^19,49^ Accordingly, adding the increasing SMRP associated with internalization symptoms and disorders to increasing SMRPs associated with school-related (underachievement and worrying about the future) and family-related (conflict with parent and severe verbal reprimand) motives can reveal a part of the complicated interactions behind increasing SMRPs among students.

Recently, perfectionism, whereby parents, educators, and students themselves highly expect to perform well in school, has become widespread in Western and East Asian countries, since good educational achievement is thought to lead to good life outcomes, including well-being, physical and mental health, and occupational status.^50^ To achieve good educational performance, parents tend to adopt attitudes with low levels of emotion and high levels of parental control or overprotection, called *affectionless control*.^50^ Both affectionless control and excessive schoolwork pressure have been established as factors associated with increased risks of internalizing symptoms and disorders, self-negative affect, and suicide among students and adolescents.^47,51,52,53,54^ In Europe, male and female students perceive increased schoolwork pressure with age; however, this pressure increases more for female students.^47^ During elementary school, female students report experiencing less pressure than male students, but the reverse is true during middle school and high school.^47^ Therefore, in Japan, the negative vicious cycle among affectionless control, perfectionism, and mental health impairment has also probably developed as a social and educational problems, similar to that in Western countries.^50,51,52,53,54^ Furthermore, the consistently increasing SMRPs among middle-school male students associated with severe verbal reprimands and underachievement, as well as that among high-school students associated with conflict with parent, underachievement, and worrying about the future, suggest the possibility that both affectionless control and excessive schoolwork pressure might also contribute to the increasing SMRPs among these male students. Therefore, the long-term increasing trends in the SMRPs of male and female students in Japan are consistent with worldwide trends.^20,21^

Governmental comprehensive suicide prevention programs via schools and communities have implemented enhanced support for students and children who have been bullied and abused. Considering the 8-fold increase in the incidence of bullying and abuse in Japan from 2007 to 2021,^55^ governmental comprehensive suicide prevention programs may have contributed to preventing SMRPs associated with bullying and abuse, since SMRPs associated with abuse and bullying did not increase from 2007 to 2022. However, governmental comprehensive suicide prevention programs have not listed addressing internalization symptoms or disorders, affectionless control, or excessive schoolwork pressure as priorities.^16^ Suppressive control of excessive schoolwork pressure and affectionless control may help to prevent suicides associated with family-related, school-related, and health-related factors among students. However, controlling internalization disorders and symptoms through interventions to prevent affectionless control or excessive schoolwork pressure may potentially violate the basic dignity of individuals and lead to unanticipated adverse reactions to the individual’s psychosocial development.^56^ Therefore, even if an effective prevention method is established, its implementation requires sufficient discussion.

Interpersonal relationships at school and in one’s family play important roles in the mental state of individuals as both risk and protective factors for suicide,^19,22^ and the impacts of these relationships transform both quantitatively and qualitatively with age.^19,22,57^ In this study, the impactable suicidal motives associated with interpersonal relationships were conflict with parents or classmates and severe verbal reprimands. Impacts of conflict with parents and severe verbal reprimands on the SMRP decreased at the transition from high school to university, whereas those of conflict with classmate increased from middle school to high school. During adolescence, individuals ordinarily decrease their time with parents and establish complicated relationships with peers by increasing their time with peers.^58^ Therefore, long-term or frequent school closures may disrupt the psychosocial development of students via interpersonal relationships.^59^ Indeed, in a recent study,^60^ 43% of students said their lives were worse, 30% said they were unchanged, and 28% said they were better during school closures; however, 15% said they were worried, 22% said they were indifferent, and 64% said they were looking forward to seeing classmates and peers again after lockdown lifted. These findings suggest that social restriction, including school closures during the pandemic, had not affected all students in the same way. Increasing time spent with parents because of school closures can easily be understood as a factor associated with increased risk of suicide among students with poor-quality relationships with their parents. Conversely, the absence of face-to-face interaction during school closure could actually be protective (at least in the short term) for students with poor-quality peer relationships or internalization symptoms. Therefore, the increasing SMRPs associated with conflict with classmate among male high-school students and university students of both sexes in 2022 indicates the possibility that the establishment of interpersonal relationships owing to resuming school attendance or normalization of the class format may be more burdensome for some students with internalization or poor-quality relationships. Further studies to explore this hypothesis may provide useful information for improving suicide prevention programs in schools.

### Limitations

This study has several limitations. Because it is impossible to collect suicide motives directly from the decedents, the suicide numbers disaggregated by suicidal motives in the SSNPA may be incorrectly estimated owing to a potential bias. However, to eliminate subjectivity as much as possible, the police investigate suicide motives based on evidence, suicide notes, official documentation (eg, medical certificates and clinical recordings), and testimony from the decedent’s family.

Although motive-unidentified suicides were homogeneous among schools and between sexes, motive-unidentified suicides might be biased toward overestimating or underestimating the results of the analysis. Despite these limitations, the SSNPA is evaluated as the most reliable governmental suicide database in Japan, since data were collected by the National Police Agency using consistent investigational methods from 2007 to 2022.

## Conclusions

The findings of this cross-sectional study suggest the importance of interaction among affectionless control, excessive schoolwork pressure, and increasing prevalence of internalization symptoms for understanding the basis for increasing student SMRPs during the late 2010s and the pandemic in Japan. The suicidal motives of students transform with advancing psychosocial developmental stages (eg, family-related motives decrease, and school-related and health-related motives increase with age). These complicated interactions among suicidal motives probably play important roles in the recent fluctuation of SMRPs in 2 increasing phases, a long-lasting increase from the late 2010s and synchronization with the pandemic outbreak. Therefore, rather than uniformly applying adolescent-based research results to school-based suicide prevention programs for middle-school, high-school, and university students, it can contribute to effective suicide prevention of students to design and implement programs that are tailored vulnerabilities of each developmental stage.

## Notes

Supplement 1. **eAppendix.** Supplemental Methods **eTable 1.** SMRPs of Middle-School Students Disaggregated by Motives and Sex During 2007-2022 **eTable 2.** SMRPs of High-School Students Disaggregated by Motives and Sex During 2007-2022 **eTable 3.** SMRPs of University Students Disaggregated by Motives and Sex During 2007-2022 **eFigure 1.** Average of SMRPs Caused by Major 7 Categorized Motive and Impactable Subcategorized Motives of Students From 2007-2022 **eTable 4.** Summary of Joinpoint of SMRP Caused by Suicidal Motives, Sex and School From 2007-2022 Analyzed by Joinpoint Regression Analysis **eFigure 2.** Age-Dependent Ratio of Prevalence of Mental Disorders Between 2017 and 2020
eReferences

Supplement 2.
Data Sharing Statement
