# Supplementary material for: Suicidal Mortality and Motives Among Middle-School, High-School, and University Students
Source: JAMA Netw Open. 2023 Aug 7;6(8):e2328144. doi: 10.1001/jamanetworkopen.2023.28144 (PMC10407687; doi:10.1001/jamanetworkopen.2023.28144)
Supplement: Supplement 1. — eAppendix. Supplemental Methods eTable 1. SMRPs of Middle-School Students Disaggregated by Motives and Sex During 2007-2022 eTable 2. SMRPs of High-School Students Disaggregated by Motives and Sex During 2007-2022 eTable 3. SMRPs of University Students Disaggregated by Motives and Sex During 2007-2022 eFigure 1. Average of SMRPs Caused by Major 7 Categorized Motive and Impactable Subcategorized Motives of Students From 2007-2022 eTable 4. Summary of Joinpoint of SMRP Caused by Suicidal Motives, Sex and School From 2007-2022 Analyzed by Joinpoint Regression Analysis eFigure 2. Age-Dependent Ratio of Prevalence of Mental Disorders Between 2017 and 2020 eReferences [file jamanetwopen-e2328144-s001.pdf]

Supplemental Online Content

Okada M, Matsumoto R, Shiroyama T, Motomura E. Suicidal mortality and motives among middle-school, high-school, and university students. *JAMA Netw Open*. 2023;6(8):e2328144. doi:10.1001/jamanetworkopen.2023.28144

eAppendix. Supplemental Methods
eTable 1. SMRPs of Middle-School Students Disaggregated by Motives and Sex During 2007-2022
eTable 2. SMRPs of High-School Students Disaggregated by Motives and Sex During 2007-2022
eTable 3. SMRPs of University Students Disaggregated by Motives and Sex During 2007-2022
eFigure 1. Average of SMRPs Caused by Major 7 Categorized Motive and Impactable Subcategorized Motives of Students From 2007-2022
eTable 4. Summary of Joinpoint of SMRP Caused by Suicidal Motives, Sex and School From 2007-2022 Analyzed by Joinpoint Regression Analysis
eFigure 2. Age-Dependent Ratio of Prevalence of Mental Disorders Between 2017 and 2020
eReferences

This supplemental material has been provided by the authors to give readers additional information about their work.

© 2023 Okada M et al. *JAMA Network Open.*

eAppendix. Supplemental Methods

Database

The “School Basic Survey” (SBS) is conducted by the Ministry of Education, Culture, Sports, Science, and Technology (MEXT) with the purpose of investigating fundamental aspects related to schools and obtaining foundational data for educational administration. SBS targets various educational institutions nationwide, including kindergartens, elementary schools, middle schools, high schools, special support school, universities and vocational schools. The main content of the survey includes the number of schools, enrolled students and graduates as of May 1st each year [1].

The Japanese government publishes two suicide statistic databases, the "Vital Statistics Registration" (VSR) of the Ministry of Health, Labor, and Welfare (MHLW) and “Suicide Statistics” (SSNPA) collected by the National Police Agency (NPA)[2-5].

VSR is a database of cause-of-death statistics in which suicide, homicide, accidental and unexplained deaths are recorded according to International Classification of Diseases (ICD) code. In Japan, only medical doctors can prepare death certificates, and the Medical Practitioners Law stipulates that abnormal death must be reported to the NPA within 24 hours[4,5].

SSNPA provides the amount values (national suicide numbers in Japan) of annual suicide numbers in each region under the jurisdiction of local police stations. The NPA must conduct physiological examinations of all corpses with abnormal causes of death to determine the cause of death[3-5]. The police must investigate the personal characteristics and background factors of each suicide case. Since it is impossible to collect suicide motives from the victims themselves, to eliminate subjectivity as much as possible, the police investigate suicide motives based on evidence, suicide notes, official documentation (e.g. medical certificates and clinical recordings) and testimony from the victim's family[3,5].

The results of this investigation discuss the different motives for suicide, and these motives are compared to previously compiled lists of motives for suicide (52 subcategories in SSNPA). The majority of suicides have diverse causes, backgrounds and complications, and occur with interactions among various factors. Therefore, the SSNPA is permitted to count multiple probable causes/motives per a suicide.

The numbers of suicides among middle-school, high-school or university students were counted according to the numbers of individuals who completed suicide when they were enrolled in middle-school, high-school or university[2,3,5-8].

The SSNPA published the annual suicide numbers disaggregated by 52 suicidal motives and sex (males and females) of students in middle-school (age: 12-15 years), high-school (age: 15-18 years) and university (age: approximately 18-22 years) during 2007-2022. Detailed explanations of the suicide motives have been described in previous reports[4,7-10], and detailed lists of categories and suicide numbers disaggregated by motive, sex and school are described in eTables 1-3.

The SSNPA did not release the number of student suicides for which the motive could not be determined. The MHLW publishes the "Basic Data on Suicide in the Region" (BDSR), which reaggregates the SSNPA data and disaggregates them into various subcategories. The BDSR also had not released number of student suicides for which the motive could not be determined until 2021. The BDSR provided for the first time in 2022 that motive-unidentified suicides numbers of students in 2022 were as followed: in middle-school males (unidentified/total: 14/73) and females (15/69), in high-school males (41/206) and females (17/146), university males (44/305) and females (24/133)[3].

Statistical analysis

Interrupted time-series analysis (ITSA) is a powerful/established method for analyzing the impacts of intervention on changing trends and discontinuity (upward/downward shifts)[11-13]. ITSA has the advantage of being able to incorporate various options, such as parametric, nonparametric regressions, seasonal variation and panel data analyses[11-13]. Based on these advantages, a number of studies detected transformed trends in suicide mortality rates during the pandemic in Japan using this approach[4,14]. However, ITSA cannot detect unknown fluctuations in the observation period.

In contrast, joinpoint regression analysis (JPRA) fits the simplest joinpoint model that the trend data allows and identifies significant points where trends change. This is a powerful statistical method for detection of unknown joinpoints (transformed trends and discontinuities)[15]. Therefore, fluctuations, including trends, discontinuity and their effect size of SMRP during 2007-2022 were analyzed by JPRA using Joinpoint Regression Program ver5.0.2 (the National Cancer Institute, Bethesda, Maryland)[15-18]. A comprehensive review of the statistics and underlying methodology applied in JPRA have been explained in a review report[15]. The detailed description of the methods used in the Joinpoint Regression Software is described in the user manual published by the National Cancer Institute (NCI)[16]. In this background, the present study analysed the fluctuations in SMRP during the 2007-2022 using JPRA. P value of <0.05 (two-tailed) were considered to indicate statistical significance.

The differences in SMRPs among schools (middle-school/high-school/university) and between sexes (male/female) from 2007-2022 were compared using linear mixed-effect model (LMM) with Scheffe’s post-hoc-test by SPSS for Windows version 27 (IBM, Armonk, NY, USA)[19,20]. When the data did not violate the assumption of sphericity ($p > 0.05$), the F value of the LMM was analyzed using sphericity-assumed degrees of freedom, whereas if the assumption of sphericity was violated ($p < 0.05$), the F value was analyzed using Greenhouse-Geisser corrected degrees of freedom. When the F value was significant ($p < 0.05$) by sphericity-assumed degrees or Greenhouse-Geisser corrected degrees of freedom, the F value of LMM were analyzed using Scheffe's post-hoc analysis [19,20]. P value of <0.05 (two-tailed) were considered to indicate statistical significance.

The differences in motives-unidentified student suicides among schools and between sexes were analyzed using the Cochran–Mantel–Haenszel test by SPSS. P value of <0.05 (two-tailed) were considered to indicate statistical significance. Cochran–Mantel–Haenszel test detected that motive-unidentified suicides were homogeneous among schools and between sexes.

© 2023 Okada M et al. *JAMA Network Open.*

eTable 1: SMRPs of Middle-School Students Disaggregated by Motives and Sex During 2007-2022.

	Males		Females	
	count	(min / max)	count	(min / max)
**Total counts of suicide motives during 2007-2022**	760	(123 / 249)	635	(14 / 83)

	Males mean ± SD	(min / max)	Females mean ± SD	(min / max)
**Population (x1000)**	1,753 ± 81	(1,639 / 1,848)	1,676 ± 79	(1,566 / 1,767)
**Family problems**	0.77 ± 0.34	(0.27 / 1.52)	0.65 ± 0.38	(0.17 / 1.47)
Conflict with parent	0.22 ± 0.12	(0.06 / 0.49)	0.32 ± 0.19	(0.11 / 0.77)
Marital conflict	0.00 ± 0.00	(0.00 / 0.00)	0.00 ± 0.00	(0.00 / 0.00)
Conflict with other family members	0.07 ± 0.08	(0.00 / 0.23)	0.07 ± 0.10	(0.00 / 0.32)
Death of family	0.02 ± 0.04	(0.00 / 0.12)	0.02 ± 0.03	(0.00 / 0.06)
Hopeless for family	0.03 ± 0.04	(0.00 / 0.12)	0.02 ± 0.03	(0.00 / 0.06)
Severe verbal reprimand	0.38 ± 0.21	(0.05 / 0.78)	0.18 ± 0.11	(0.00 / 0.32)
Stress of raising children	0.00 ± 0.00	(0.00 / 0.00)	0.00 ± 0.00	(0.00 / 0.00)
Physical and/or verbal abuse	0.00 ± 0.01	(0.00 / 0.06)	0.00 ± 0.00	(0.00 / 0.00)
Exhaustion from caring for infirm family	0.00 ± 0.01	(0.00 / 0.05)	0.00 ± 0.00	(0.00 / 0.00)
**Health problems**	0.29 ± 0.17	(0.05 / 0.73)	0.39 ± 0.32	(0.06 / 1.02)
Physical illness	0.04 ± 0.06	(0.00 / 0.18)	0.04 ± 0.04	(0.00 / 0.13)
Depression	0.09 ± 0.07	(0.00 / 0.23)	0.13 ± 0.14	(0.00 / 0.51)
Schizophrenia	0.01 ± 0.02	(0.00 / 0.06)	0.01 ± 0.03	(0.00 / 0.06)
Alcoholism	0.00 ± 0.00	(0.00 / 0.00)	0.00 ± 0.00	(0.00 / 0.00)
Drug abuse	0.00 ± 0.00	(0.00 / 0.00)	0.00 ± 0.00	(0.00 / 0.00)
Other mental illness	0.11 ± 0.07	(0.00 / 0.28)	0.18 ± 0.23	(0.00 / 0.83)
Physical disability	0.01 ± 0.02	(0.00 / 0.06)	0.01 ± 0.03	(0.00 / 0.13)
**Economic problems**	0.00 ± 0.02	(0.00 / 0.06)	0.00 ± 0.00	(0.00 / 0.00)
Bankruptcy	0.00 ± 0.00	(0.00 / 0.00)	0.00 ± 0.00	(0.00 / 0.00)
Business struggling	0.00 ± 0.00	(0.00 / 0.00)	0.00 ± 0.00	(0.00 / 0.00)
Unemployment	0.00 ± 0.00	(0.00 / 0.00)	0.00 ± 0.00	(0.00 / 0.00)
Inability to find employment	0.00 ± 0.00	(0.00 / 0.00)	0.00 ± 0.00	(0.00 / 0.00)
Economic hardships	0.00 ± 0.00	(0.00 / 0.00)	0.00 ± 0.00	(0.00 / 0.00)
Overloaded with debt	0.00 ± 0.00	(0.00 / 0.00)	0.00 ± 0.00	(0.00 / 0.00)
Assumption of excessive debt	0.00 ± 0.00	(0.00 / 0.00)	0.00 ± 0.00	(0.00 / 0.00)
Debt (other)	0.00 ± 0.00	(0.00 / 0.00)	0.00 ± 0.00	(0.00 / 0.00)
Harassment by debt-collectors	0.00 ± 0.00	(0.00 / 0.00)	0.00 ± 0.00	(0.00 / 0.00)
Suicide for death benefit	0.00 ± 0.00	(0.00 / 0.00)	0.00 ± 0.00	(0.00 / 0.00)
**Employment problems**	0.00 ± 0.00	(0.00 / 0.00)	0.00 ± 0.00	(0.00 / 0.00)
Failure at work	0.00 ± 0.00	(0.00 / 0.00)	0.00 ± 0.00	(0.00 / 0.00)
Inter-personal relations at work	0.00 ± 0.00	(0.00 / 0.00)	0.00 ± 0.00	(0.00 / 0.00)
Trouble adjusting to changing work environment	0.00 ± 0.00	(0.00 / 0.00)	0.00 ± 0.00	(0.00 / 0.00)
Work-related fatigue	0.00 ± 0.00	(0.00 / 0.00)	0.00 ± 0.00	(0.00 / 0.00)
**Romantic problems**	0.09 ± 0.09	(0.00 / 0.30)	0.08 ± 0.06	(0.00 / 0.17)
Marital problems	0.00 ± 0.00	(0.00 / 0.00)	0.00 ± 0.00	(0.00 / 0.00)
Heartbreak	0.06 ± 0.07	(0.00 / 0.23)	0.04 ± 0.04	(0.00 / 0.12)
Extra-marital affair	0.00 ± 0.00	(0.00 / 0.00)	0.00 ± 0.00	(0.00 / 0.00)
Conflict in relationship	0.03 ± 0.04	(0.00 / 0.12)	0.03 ± 0.04	(0.00 / 0.12)
**School problems**	1.33 ± 0.42	(0.97 / 2.62)	1.10 ± 0.62	(0.34 / 2.62)
Entrance examination problems	0.18 ± 0.15	(0.05 / 0.61)	0.12 ± 0.12	(0.00 / 0.45)
Worrying about future	0.26 ± 0.17	(0.06 / 0.79)	0.18 ± 0.19	(0.00 / 0.77)
Underachievement	0.40 ± 0.13	(0.22 / 0.79)	0.22 ± 0.16	(0.00 / 0.57)
Interpersonal relations with teachers	0.05 ± 0.06	(0.00 / 0.18)	0.03 ± 0.05	(0.00 / 0.13)
Bullying	0.08 ± 0.06	(0.00 / 0.22)	0.06 ± 0.05	(0.00 / 0.19)
Conflict with classmate	0.15 ± 0.10	(0.00 / 0.34)	0.29 ± 0.13	(0.12 / 0.57)
**Others problems**	0.25 ± 0.20	(0.00 / 0.79)	0.21 ± 0.18	(0.00 / 0.57)
Public disclosure of crime	0.04 ± 0.05	(0.00 / 0.12)	0.00 ± 0.02	(0.00 / 0.06)
Crime victim	0.00 ± 0.02	(0.00 / 0.06)	0.00 ± 0.00	(0.00 / 0.00)
Copycat suicide	0.01 ± 0.03	(0.00 / 0.11)	0.00 ± 0.02	(0.00 / 0.06)
Loneliness	0.04 ± 0.08	(0.00 / 0.30)	0.06 ± 0.08	(0.00 / 0.26)
Neighborhood problems	0.00 ± 0.00	(0.00 / 0.00)	0.00 ± 0.00	(0.00 / 0.00)
**Total**	**2.74 ± 1.01**	**(1.46 / 5.79)**	**2.42 ± 1.38**	**(0.81 / 5.30)**

© 2023 Okada M et al. *JAMA Network Open.*

eTable 2: SMRPs of High-School Students Disaggregated by Motives and Sex During 2007-2022.

	Males count	Males (min / max)	Females count	Females (min / max)
**Total counts of suicide motives during 2007-2022**	2376	(123 / 249)	1566	(66 / 135)

	Males mean ± SD	Males (min / max)	Females mean ± SD	Females (min / max)
**Population (x1000)**	1,648 ± 68	(1,499 / 1,724)	1,615 ± 60	(1,458 / 1,674)
**Family problems**	1.42 ± 0.49	(0.89 / 2.53)	1.06 ± 0.46	(0.36 / 1.99)
Conflict with parent	0.59 ± 0.22	(0.24 / 0.96)	0.44 ± 0.25	(0.12 / 0.87)
Marital conflict	0.00 ± 0.00	(0.00 / 0.00)	0.01 ± 0.02	(0.00 / 0.07)
Conflict with other family members	0.13 ± 0.15	(0.00 / 0.45)	0.17 ± 0.09	(0.00 / 0.33)
Death of family	0.06 ± 0.05	(0.00 / 0.13)	0.06 ± 0.07	(0.00 / 0.21)
Hopeless for family	0.06 ± 0.05	(0.00 / 0.18)	0.04 ± 0.05	(0.00 / 0.20)
Severe verbal reprimand	0.37 ± 0.22	(0.12 / 1.00)	0.23 ± 0.13	(0.06 / 0.48)
Stress of raising children	0.00 ± 0.00	(0.00 / 0.00)	0.00 ± 0.00	(0.00 / 0.00)
Physical and/or verbal abuse	0.02 ± 0.03	(0.00 / 0.07)	0.02 ± 0.04	(0.00 / 0.14)
Exhaustion from caring for infirm family	0.00 ± 0.00	(0.00 / 0.00)	0.00 ± 0.00	(0.00 / 0.00)
**Health problems**	1.64 ± 0.38	(1.03 / 2.33)	2.06 ± 0.95	(1.11 / 4.53)
Physical illness	0.20 ± 0.09	(0.06 / 0.39)	0.12 ± 0.09	(0.00 / 0.30)
Depression	0.59 ± 0.27	(0.00 / 1.06)	0.88 ± 0.43	(0.31 / 1.99)
Schizophrenia	0.20 ± 0.14	(0.00 / 0.48)	0.29 ± 0.12	(0.06 / 0.48)
Alcoholism	0.00 ± 0.00	(0.00 / 0.00)	0.00 ± 0.00	(0.00 / 0.00)
Drug abuse	0.01 ± 0.03	(0.00 / 0.12)	0.01 ± 0.03	(0.00 / 0.07)
Other mental illness	0.51 ± 0.24	(0.12 / 1.20)	0.67 ± 0.54	(0.18 / 2.08)
Physical disability	0.03 ± 0.05	(0.00 / 0.20)	0.03 ± 0.04	(0.00 / 0.14)
**Economic problems**	0.13 ± 0.11	(0.00 / 0.40)	0.05 ± 0.04	(0.00 / 0.14)
Bankruptcy	0.00 ± 0.00	(0.00 / 0.00)	0.00 ± 0.00	(0.00 / 0.00)
Business struggling	0.00 ± 0.00	(0.00 / 0.00)	0.00 ± 0.00	(0.00 / 0.00)
Unemployment	0.00 ± 0.00	(0.00 / 0.00)	0.00 ± 0.00	(0.00 / 0.00)
Inability to find employment	0.05 ± 0.07	(0.00 / 0.24)	0.02 ± 0.03	(0.00 / 0.12)
Economic hardships	0.02 ± 0.04	(0.00 / 0.13)	0.02 ± 0.03	(0.00 / 0.06)
Overloaded with debt	0.00 ± 0.02	(0.00 / 0.06)	0.00 ± 0.00	(0.00 / 0.00)
Assumption of excessive debt	0.00 ± 0.00	(0.00 / 0.00)	0.00 ± 0.00	(0.00 / 0.00)
Debt (other)	0.02 ± 0.04	(0.00 / 0.13)	0.00 ± 0.00	(0.00 / 0.00)
Harassment by debt-collectors	0.00 ± 0.01	(0.00 / 0.06)	0.00 ± 0.00	(0.00 / 0.00)
Suicide for death benefit	0.00 ± 0.00	(0.00 / 0.00)	0.00 ± 0.00	(0.00 / 0.00)
**Employment problems**	0.03 ± 0.04	(0.00 / 0.12)	0.03 ± 0.03	(0.00 / 0.07)
Failure at work	0.00 ± 0.02	(0.00 / 0.06)	0.00 ± 0.02	(0.00 / 0.06)
Inter-personal relations at work	0.01 ± 0.02	(0.00 / 0.06)	0.01 ± 0.03	(0.00 / 0.07)
Trouble adjusting to changing work environment	0.00 ± 0.00	(0.00 / 0.00)	0.00 ± 0.02	(0.00 / 0.06)
Work-related fatigue	0.00 ± 0.00	(0.00 / 0.00)	0.00 ± 0.00	(0.00 / 0.00)
**Romantic problems**	0.78 ± 0.19	(0.49 / 1.20)	0.54 ± 0.21	(0.24 / 0.87)
Marital problems	0.00 ± 0.00	(0.00 / 0.00)	0.00 ± 0.00	(0.00 / 0.00)
Heartbreak	0.52 ± 0.20	(0.24 / 0.93)	0.25 ± 0.15	(0.06 / 0.51)
Extra-marital affair	0.00 ± 0.02	(0.00 / 0.07)	0.01 ± 0.02	(0.00 / 0.07)
Conflict in relationship	0.21 ± 0.13	(0.00 / 0.42)	0.22 ± 0.13	(0.00 / 0.48)
**School problems**	4.17 ± 1.34	(2.88 / 8.34)	1.94 ± 0.90	(1.15 / 4.39)
Entrance examination problems	0.53 ± 0.21	(0.18 / 1.00)	0.20 ± 0.10	(0.00 / 0.39)
Worrying about future	1.17 ± 0.33	(0.77 / 1.81)	0.49 ± 0.26	(0.24 / 1.31)
Underachievement	1.32 ± 0.51	(0.82 / 2.87)	0.36 ± 0.22	(0.18 / 1.03)
Interpersonal relations with teachers	0.08 ± 0.08	(0.00 / 0.33)	0.06 ± 0.06	(0.00 / 0.18)
Bullying	0.06 ± 0.06	(0.00 / 0.18)	0.08 ± 0.10	(0.00 / 0.27)
Conflict with classmate	0.43 ± 0.19	(0.24 / 1.07)	0.41 ± 0.29	(0.18 / 1.30)
**Others problems**	0.92 ± 0.34	(0.47 / 1.73)	0.48 ± 0.24	(0.06 / 1.03)
Public disclosure of crime	0.09 ± 0.07	(0.00 / 0.26)	0.04 ± 0.07	(0.00 / 0.27)
Crime victim	0.00 ± 0.01	(0.00 / 0.06)	0.02 ± 0.03	(0.00 / 0.07)
Copycat suicide	0.02 ± 0.03	(0.00 / 0.07)	0.03 ± 0.04	(0.00 / 0.12)
Loneliness	0.25 ± 0.10	(0.06 / 0.42)	0.17 ± 0.08	(0.00 / 0.34)
Neighborhood problems	0.00 ± 0.00	(0.00 / 0.00)	0.00 ± 0.02	(0.00 / 0.06)
**Total**	**9.09 ± 2.40**	**(7.33 / 16.61)**	**6.14 ± 2.39**	**(4.01 / 12.69)**

© 2023 Okada M et al. *JAMA Network Open.*

eTable 3: SMRPs of University Students Disaggregated by Motives and Sex During 2007-2022.

	Males count	Males (min / max)	Females count	Females (min / max)
**Total counts of suicide motives during 2007-2022**	5179	(221 / 397)	1880	(86 / 158)

	Males mean ± SD	Males (min / max)	Females mean ± SD	Females (min / max)
**Population (x1000)**	1,653 ± 33	(1,621 / 1,702)	1,229 ± 57	(1,127 / 1,304)
**Family problems**	1.43 ± 0.37	(0.80 / 2.03)	0.80 ± 0.21	(0.36 / 1.08)
Conflict with parent	0.45 ± 0.23	(0.18 / 0.98)	0.27 ± 0.12	(0.08 / 0.47)
Marital conflict	0.02 ± 0.03	(0.00 / 0.06)	0.02 ± 0.04	(0.00 / 0.09)
Conflict with other family members	0.16 ± 0.08	(0.06 / 0.37)	0.15 ± 0.10	(0.00 / 0.32)
Death of family	0.09 ± 0.06	(0.00 / 0.24)	0.07 ± 0.07	(0.00 / 0.23)
Hopeless for family	0.15 ± 0.10	(0.00 / 0.37)	0.10 ± 0.08	(0.00 / 0.26)
Severe verbal reprimand	0.31 ± 0.15	(0.12 / 0.65)	0.10 ± 0.10	(0.00 / 0.33)
Stress of raising children	0.00 ± 0.01	(0.00 / 0.06)	0.02 ± 0.04	(0.00 / 0.08)
Physical and/or verbal abuse	0.00 ± 0.02	(0.00 / 0.06)	0.00 ± 0.00	(0.00 / 0.00)
Exhaustion from caring for infirm family	0.01 ± 0.02	(0.00 / 0.06)	0.00 ± 0.00	(0.00 / 0.00)
**Health problems**	4.65 ± 1.24	(2.70 / 7.29)	3.71 ± 0.94	(2.65 / 5.24)
Physical illness	0.38 ± 0.15	(0.12 / 0.76)	0.22 ± 0.10	(0.08 / 0.35)
Depression	2.35 ± 0.93	(1.11 / 4.33)	2.14 ± 0.88	(1.22 / 3.94)
Schizophrenia	0.58 ± 0.24	(0.25 / 1.13)	0.37 ± 0.15	(0.15 / 0.67)
Alcoholism	0.01 ± 0.02	(0.00 / 0.06)	0.00 ± 0.02	(0.00 / 0.08)
Drug abuse	0.01 ± 0.02	(0.00 / 0.06)	0.02 ± 0.04	(0.00 / 0.09)
Other mental illness	1.07 ± 0.23	(0.67 / 1.54)	0.83 ± 0.25	(0.53 / 1.31)
Physical disability	0.06 ± 0.07	(0.00 / 0.24)	0.02 ± 0.03	(0.00 / 0.09)
**Economic problems**	2.09 ± 0.68	(1.17 / 3.47)	0.53 ± 0.24	(0.09 / 1.00)
Bankruptcy	0.00 ± 0.00	(0.00 / 0.00)	0.00 ± 0.00	(0.00 / 0.00)
Business struggling	0.01 ± 0.02	(0.00 / 0.06)	0.00 ± 0.00	(0.00 / 0.00)
Unemployment	0.00 ± 0.00	(0.00 / 0.00)	0.00 ± 0.00	(0.00 / 0.00)
Inability to find employment	1.30 ± 0.55	(0.61 / 2.51)	0.38 ± 0.21	(0.00 / 0.77)
Economic hardships	0.16 ± 0.10	(0.00 / 0.37)	0.02 ± 0.04	(0.00 / 0.08)
Overloaded with debt	0.10 ± 0.08	(0.00 / 0.25)	0.01 ± 0.03	(0.00 / 0.08)
Assumption of excessive debt	0.00 ± 0.00	(0.00 / 0.00)	0.00 ± 0.00	(0.00 / 0.00)
Debt (other)	0.12 ± 0.10	(0.00 / 0.37)	0.01 ± 0.04	(0.00 / 0.15)
Harassment by debt-collectors	0.00 ± 0.00	(0.00 / 0.00)	0.00 ± 0.00	(0.00 / 0.00)
Suicide for death benefit	0.00 ± 0.00	(0.00 / 0.00)	0.00 ± 0.00	(0.00 / 0.00)
**Employment problems**	0.21 ± 0.16	(0.00 / 0.47)	0.07 ± 0.09	(0.00 / 0.23)
Failure at work	0.04 ± 0.04	(0.00 / 0.12)	0.01 ± 0.02	(0.00 / 0.09)
Inter-personal relations at work	0.04 ± 0.06	(0.00 / 0.18)	0.02 ± 0.05	(0.00 / 0.15)
Trouble adjusting to changing work environment	0.02 ± 0.03	(0.00 / 0.06)	0.00 ± 0.00	(0.00 / 0.00)
Work-related fatigue	0.02 ± 0.04	(0.00 / 0.12)	0.03 ± 0.05	(0.00 / 0.17)
**Romantic problems**	1.23 ± 0.37	(0.49 / 2.01)	1.00 ± 0.29	(0.56 / 1.77)
Marital problems	0.04 ± 0.07	(0.00 / 0.24)	0.02 ± 0.04	(0.00 / 0.09)
Heartbreak	0.77 ± 0.28	(0.25 / 1.23)	0.43 ± 0.13	(0.23 / 0.67)
Extra-marital affair	0.01 ± 0.03	(0.00 / 0.12)	0.05 ± 0.07	(0.00 / 0.25)
Conflict in relationship	0.34 ± 0.17	(0.00 / 0.71)	0.43 ± 0.17	(0.00 / 0.67)
**School problems**	8.28 ± 1.35	(6.46 / 10.60)	2.74 ± 0.76	(1.68 / 4.37)
Entrance examination problems	0.13 ± 0.09	(0.00 / 0.37)	0.03 ± 0.05	(0.00 / 0.15)
Worrying about future	3.03 ± 0.59	(2.03 / 3.96)	1.05 ± 0.34	(0.48 / 1.84)
Underachievement	3.69 ± 0.64	(2.64 / 4.55)	0.88 ± 0.40	(0.24 / 1.67)
Interpersonal relations with teachers	0.10 ± 0.10	(0.00 / 0.37)	0.04 ± 0.07	(0.00 / 0.25)
Bullying	0.03 ± 0.04	(0.00 / 0.12)	0.01 ± 0.02	(0.00 / 0.08)
Conflict with classmate	0.47 ± 0.21	(0.25 / 1.04)	0.35 ± 0.22	(0.08 / 0.69)
**Others problems**	1.67 ± 0.46	(1.04 / 2.77)	0.72 ± 0.29	(0.16 / 1.16)
Public disclosure of crime	0.13 ± 0.06	(0.06 / 0.25)	0.00 ± 0.02	(0.00 / 0.08)
Crime victim	0.01 ± 0.02	(0.00 / 0.06)	0.00 ± 0.00	(0.00 / 0.00)
Copycat suicide	0.03 ± 0.03	(0.00 / 0.06)	0.03 ± 0.04	(0.00 / 0.09)
Loneliness	0.44 ± 0.29	(0.12 / 1.23)	0.21 ± 0.17	(0.00 / 0.70)
Neighborhood problems	0.01 ± 0.02	(0.00 / 0.06)	0.01 ± 0.02	(0.00 / 0.09)
**Total**	**19.55 ± 3.04**	**(13.57 / 24.09)**	**9.57 ± 1.79**	**(6.89 / 12.16)**

© 2023 Okada M et al. *JAMA Network Open.*

eFigure 1: Average of SMRPs Caused by Major 7 Categorized Motive and Impactable Subcategorized Motives of Students From 2007-2022.

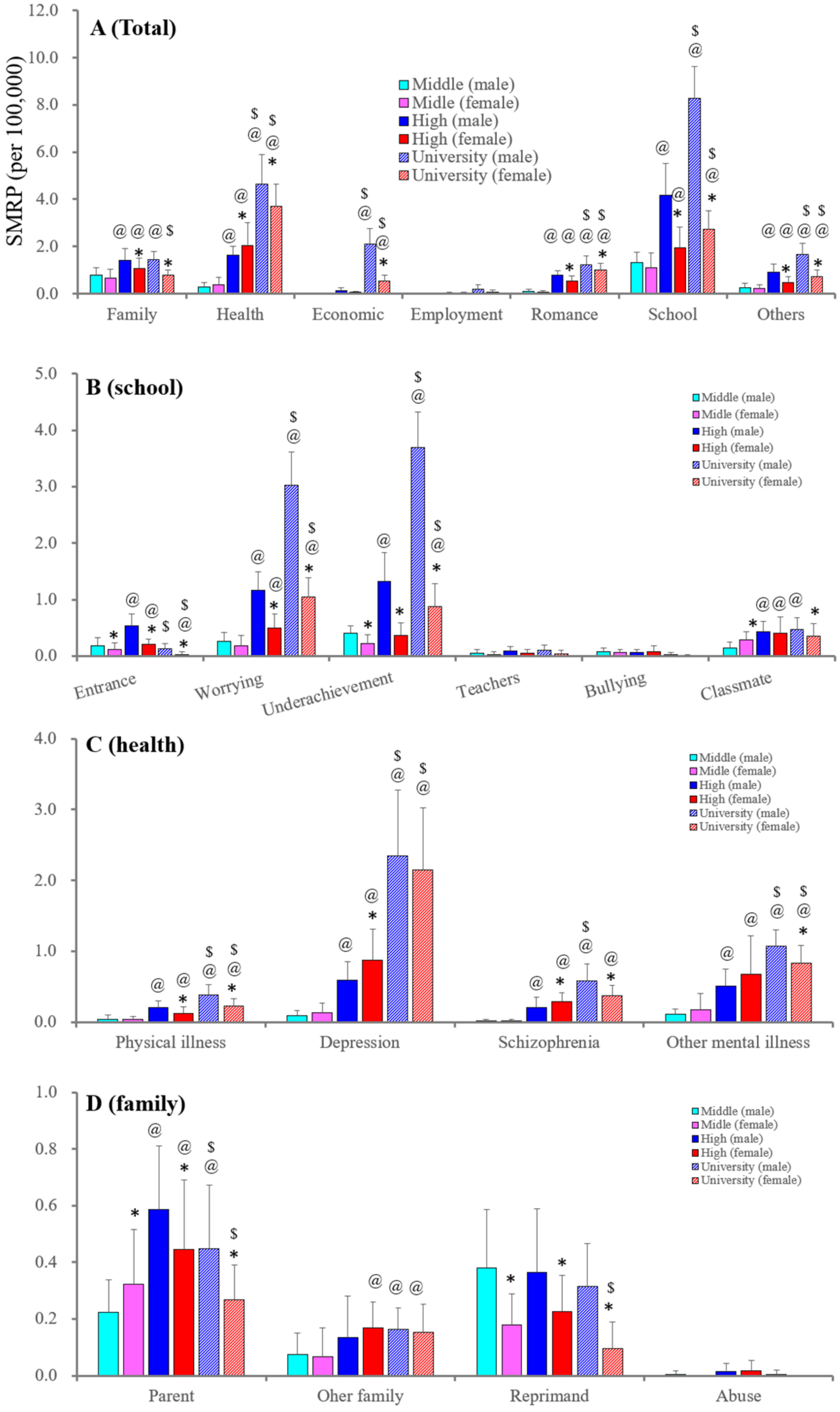

SMRPs caused by major 7 categorized motives (A), subcategorized motives in in school- (B), health- (C) and family-related motives (D) of males and females students in middle-school, high-school and university. Ordinate indicate mean±SD of SMRs from 2007-2022. *: P<0.05 relative to males of same school group, @:P<0.05 relative to middle-school of same sex, $:P<0.05 relative to high-school of same sex, using linear mixed-effect model with Scheffe's post-hoc test.

© 2023 Okada M et al. *JAMA Network Open.*

eTable 4: Summary of Joinpoint of SMRP Caused by Suicidal Motives, Sex and School From 2007-2022 Analyzed by Joinpoint Regression Analysis

		Males					Females				
Cohort		period	□	(95 % CI)	T	P	period	□	(95 % CI)	T	P
Total	middle	2007-2021	0.04	(0.01 - 0.06)	3.31	0.006**	2007-2014	0.02	(-0.10 - 0.14)	0.28	0.786
		2021-2022	0.58	(-)	-	-	2014-2022	0.17	(0.07 - 0.26)	3.43	0.001**
	high	2007-2018	0.01	(-0.01 - 0.03)	0.87	0.404	2007-2019	-0.09	(-0.05 - 0.03)	0.44	0.668
		2018-2022	0.14	(0.06 - 0.23)	3.28	0.007**	2019-2022	0.18	(0.07 - 0.30)	3.19	0.009 **
	university	2007-2009	0.11	(-0.16 - 0.37)	0.80	0.446	2007-2010	0.08	(-0.09 - 0.25)	0.89	0.400
		2009-2021	-0.04	(-0.05 - -0.02)	-3.93	0.003**	2010-2016	-0.09	(-0.17 - -0.01)	-2.33	0.048*
		2021-2022	0.46	(-)	-	-	2016-2022	0.09	(0.03 - 0.15)	3.13	0.014*
School	middle	2007-2015	0.067	(0.04 - 0.10)	4.44	0.002**	2007-2009	0.59	(-0.26 - 1.44)	1.36	0.210
		2015-2020	-0.067	(-0.15 - 0.02)	-1.55	0.160	2009-2012	-0.29	(-1.14 - 0.56)	-0.68	0.517
		2020-2022	0.327	(0.06 - 0.60)	2.37	0.045*	2012-2022	0.15	(0.08 - 0.21)	4.31	0.003**
	high	2007-2016	0.00	(-0.04 - 0.04)	-0.15	0.881	2007-2019	-0.01	(-0.04 - 0.04)	-0.15	0.881
		2016-2022	0.11	(0.04 - 0.18)	3.13	0.010*	2019-2022	0.11	(0.04 - 0.18)	3.13	0.009**
	university	2007-2012	0.06	(0.03 - 0.09)	3.71	0.006**	2007-2011	0.14	(-0.02 - 0.30)	1.76	0.116
		2012-2020	-0.07	(-0.08 - -0.05)	-6.98	0.001**	2011-2016	-0.14	(-0.30 - 0.02)	-1.73	0.122
		2020-2022	0.25	(0.12 - 0.39)	3.63	0.007**	2016-2022	0.13	(0.04 - 0.21)	2.97	0.018*
(under-achievement)	middle	2007-2021	0.03	(0.00 - 0.01)	2.75	0.018**	2007-2022	0.02	(0.01 - 0.04)	3.96	0.001**
		2021-2022	0.54	(-)	-	-					
	high	2007-2012	0.06	(-0.04 - 0.17)	1.18	0.274	2007-2019	0.03	(-0.03 - 0.10)	0.96	0.356
		2012-2016	-0.04	(-0.27 - 0.20)	-0.30	0.771	2019-2022	0.29	(-0.27 - 0.84)	1.01	0.333
		2016-2022	0.13	(0.05 - 0.21)	3.29	0.011*					
	university	2007-2009	0.23	(-0.12 - 0.58)	1.30	0.229	2007-2013	0.13	(-0.05 - 0.32)	1.40	0.195
		2009-2019	-0.05	(-0.08 - -0.02)	-3.03	0.016*	2013-2015	-0.66	(-)	-	-
		2019-2022	0.11	(-0.07 - 0.28)	1.22	0.257	2015-2022	0.19	(0.04 - 0.34)	2.51	0.033*
(worry about future)	middle	2007-2021	0.00	(-0.01 - 0.01)	0.36	0.725	2007-2021	0.01	(0.00 - 0.03)	1.98	0.071
		2021-2022	0.56	(-)	-	-	2021-2022	0.54	(-)	-	-
	high	2007-2011	0.08	(-0.08 - 0.24)	1.03	0.334	2007-2008	0.80	(-)	-	-
		2011-2016	-0.05	(-0.21 - 0.11)	-0.61	0.560	2008-2018	-0.03	(-0.16 - 0.09)	-0.55	0.598
		2016-2022	0.12	(0.03 - 0.20)	2.75	0.025*	2018-2022	-0.38	(-0.80 - 0.04)	-1.77	0.110
							Jump	1.68	(0.03 - 3.33)	2.35	0.046*
	university	2007-2011	0.12	(0.02 - 0.22)	2.32	0.049*	2007-2014	0.04	(-0.07 - 0.14)	0.73	0.482
		2011-2019	-0.08	(-0.12 - -0.03)	-3.46	0.009**	2014-2016	-0.22	(-)	-	-
		2019-2022	0.15	(-0.01 - 0.31)	1.82	0.106	2016-2022	0.13	(0.00 - 0.27)	1.97	0.080
(conflict with classmate)	middle	2007-2012	-0.01	(-0.08 - 0.05)	-0.38	0.713	2007-2013	0.02	(-0.01 - 0.05)	1.28	0.237
		2012-2014	0.07	(-)	-	-	2013-2017	-0.02	(-0.10 - 0.06)	-0.43	0.679
		2014-2022	-0.02	(-0.05 - 0.01)	-1.22	0.254	2017-2022	0.07	(0.03 - 0.11)	3.78	0.005**
	high	2007-2016	-0.03	(-0.10 - 0.04)	-0.78	0.455	2007-2017	0.00	(-0.06 - 0.06)	-0.03	0.974
		2016-2020	0.05	(-0.34 - 0.44)	0.26	0.804	2017-2022	0.29	(0.11 - 0.46)	3.23	0.008**
		2020-2022	0.40	(-0.38 - 1.18)	1.00	0.346					
	university	2007-2015	0.07	(0.00 - 0.14)	1.85	0.098	2007-2014	0.00	(-0.36 - 0.36)	0.00	0.999
		2015-2021	-0.17	(-0.32 - -0.02)	-2.19	0.056	2014-2021	-0.15	(-0.60 - 0.30)	-0.65	0.532
		2021-2022	1.54	(-)	-	-	2021-2022	1.82	(-)	-	-
Health	middle	2007-2016	0.19	(0.12 - 0.25)	5.64	0.001**	2007-2009	0.44	(-0.37 -1.25)	1.07	0.317
		2016-2020	-0.36	(-0.71 - 0.00)	-1.95	0.087	2009-2012	-0.55	(-1.36 -0.26)	-1.34	0.218
		2020-2022	0.95	(0.24 - 1.67)	2.61	0.031*	2012-2022	0.27	(0.20 -0.33)	8.31	0.001**
	high	2007-2018	-0.03	(-0.06 - 0.00)	-1.94	0.079	2007-2017	-0.04	(-0.08 - -0.01)	-2.22	0.048*
		2018-2022	0.17	(0.02 - 0.32)	2.27	0.044*	2017-2022	0.25	(0.14 - 0.37)	4.45	0.001**
	university	2007-2018	-0.08	(-0.11 - -0.06)	-6.38	0.001**	2007-2016	-0.09	(-0.12 - -0.06)	-5.63	0.000**
		2018-2022	0.11	(0.04 - 0.18)	3.05	0.011*	2016-2022	0.09	(0.03 - 0.14)	3.03	0.011*
(depression)	middle	2007-2016	0.01	(0.00 - 0.03)	1.81	0.107	2007-2010	0.07	(-0.10 - 0.23)	0.80	0.448
		2016-2019	-0.04	(-0.20 - 0.12)	-0.49	0.640	2010-2013	-0.11	(-0.44 - 0.22)	-0.66	0.528
		2019-2022	0.05	(-0.03 - 0.13)	1.20	0.264	2013-2022	0.04	(0.01 - 0.07)	2.60	0.031*
	high	2007-2022	-0.13	(-0.22 - -0.03)	-2.88	0.012*	2007-2022	-0.07	(-0.12 - -0.01)	-2.38	0.032*
		Jump	0.56	(-0.14 - 2.90)	1.96	0.072*	Jump	1.27	(0.62 - 1.92)	4.23	0.001**
	university	2007-2017	-0.11	(-0.16 - -0.06)	-4.13	0.002**	2007-2015	-0.15	(-0.20 - -0.10)	-5.56	0.001**
		2017-2022	0.10	(-0.05 - 0.25)	1.33	0.211	2015-2022	0.08	(0.01 - 0.14)	2.29	0.042*
(other mental illness)	middle	2007-2022	0.01	(0.00 - 0.01)	1.75	0.102	2007-2013	-0.01	(-0.09 - 0.07)	-0.20	0.848
							2013-2022	0.06	(0.01 - 0.10)	2.51	0.029*
	high	2007-2010	-0.23	(-0.75 - 0.30)	-0.84	0.420	2007-2022	0.06	(0.01 - 0.10)	2.46	0.027*
		2010-2022	0.09	(0.03 - 0.16)	2.98	0.013*	Jump	0.86	(0.19 - 1.52)	2.78	0.016*
	university	2007-2020	0.01	(-0.02 - 0.04)	0.46	0.651	2007-2009	0.25	(-0.24 - 0.74)	1.01	0.342
		2020-2022	0.22	(-0.32 - 0.76)	0.78	0.449	2009-2016	-0.05	(-0.13 - 0.04)	-1.13	0.291
Family	middle	2007-2022	0.06	(0.02 - 0.10)	3.09	0.008**	2007-2009	0.58	(-0.57 - 1.72)	0.98	0.356
							2009-2011	-0.12	(-1.27 - 1.03)	-0.20	0.846
							2011-2022	0.14	(0.05 - 0.23)	2.98	0.018**
	high	2007-2010	0.15	(-0.02 - 0.32)	1.71	0.126	2007-2011	0.25	(0.05 - 0.44)	2.43	0.041*

© 2023 Okada M et al. *JAMA Network Open.*

		2010-2013	-0.14	(-0.49 - 0.20)	-0.81	0.444	2011-2013	-0.08	(-0.71 - 0.54)	-0.26	0.799
		2013-2022	0.11	(0.08 - 0.14)	6.77	0.001**	2013-2022	0.10	(0.05 - 0.17)	2.88	0.021*
	university	2007-2016	0.04	(-0.01 - 0.10)	1.49	0.169	2007-2009	0.589	(-)		
		2016-2021	-0.09	(-0.29 - 0.10)	-0.93	0.375	2009-2011	-0.024	(-0.10 - 0.05)	-0.621	0.550
		2021-2022	0.65	(-)	-	-	2011-2022	0.063	(-0.09 - 0.22)	0.808	0.440
(conflict with parent)	middle	2007-2020	0.01	(-0.08 - 0.10)	0.21	0.839	2007-2020	0.12	(0.05 - 0.18)	3.57	0.004**
		2020-2022	0.49	(-1.18 - 2.16)	0.57	0.578	2020-2022	-0.21	(-1.45 - 1.03)	-0.34	0.743
	high	2007-2010	0.15	(-0.27 - 0.57)	0.72	0.494	2007-2011	0.33	(0.17 - 0.49)	4.14	0.003**
		2010-2014	-0.10	(-0.52 - 0.32)	-0.45	0.665	2011-2014	-0.06	(-0.55 - 0.44)	-0.23	0.824
		2014-2022	0.12	(0.02 - 0.21)	2.47	0.039*	2014-2022	0.14	(0.08 - 0.19)	4.94	0.001**
	university	2007-2017	0.02	(-0.06 - 0.09)	0.40	0.696	2007-2011	0.31	(-0.10 - 0.73)	1.47	0.174
		2017-2022	0.16	(-0.06 - 0.37)	1.43	0.181	2011-2013	-0.64	(-)	-	-
							2013-2022	0.15	(0.03 - 0.27)	2.40	0.040*
(severe verbal reprimand)	middle	2007-2022	0.02	(0.00 - 0.04)	2.40	0.031*	2007-2022	0.02	(0.01 - 0.03)	3.62	0.003**
	high	2007-2021	0.04	(-0.03 - 0.10)	1.11	0.290	2007-2010	0.53	(-0.32 - 1.37)	1.23	0.251
		2021-2022	0.99	(-)	-	-	2010-2012	-0.44	(-)	-	-
							2012-2022	0.05	(-0.08 - 0.18)	0.78	0.456
	university	2007-2014	0.02	(-0.04 - 0.08)	0.60	0.562	2007-2012	0.04	(-0.01 - 0.08)	1.57	0.145
		2014-2022	-0.02	(-0.07 - 0.04)	-0.59	0.568	2012-2022	-0.01	(-0.02 - 0.01)	-1.12	0.287

© 2023 Okada M et al. *JAMA Network Open.*

eFigure 2: Age-Dependent Ratio of Prevalence of Mental Disorders Between 2017 and 2020.

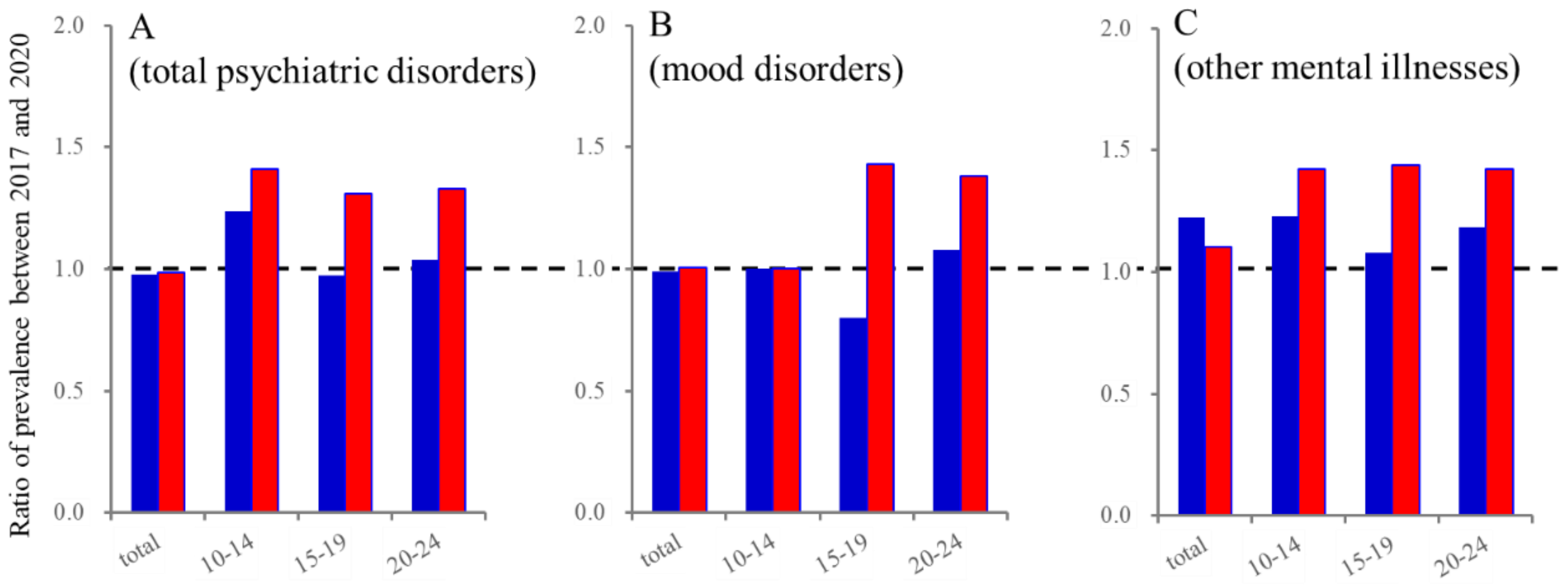

Ratio of prevalence of total psychiatric disorders (A), mood disorders (B) and other mental illnesses (C) between 2017 and 2020 (2020/2017) obtained from the "Patients Survey"[21]. Ordinates indicate the ratio of prevalence (2020/2017) of males (blue bars) and females (red bars), and abscissas indicate the range of age.

eReferences

1. MEXT. School Basic Survey. 2022; https://www.e-stat.go.jp/en/statistics/00400001. Accessed 2023.1.31, 2023.
2. NPA. Suicide Statistics (SSNPA). 2023; https://www.npa.go.jp/publications/statistics/safetylife/jisatsu.html. Accessed 2023.3.30, 2023.
3. MHLW. Basic data on suicide in the region. 2023; https://www.mhlw.go.jp/stf/seisakunitsuite/bunya/0000140901.html. Accessed 2023.1.31, 2023.
4. Koda M, Harada N, Eguchi A, Nomura S, Ishida Y. Reasons for Suicide During the COVID-19 Pandemic in Japan. *JAMA Netw Open.* 2022;5(1):e2145870.
5. MHLW. 2021 White Paper on Suicide Prevention. 2022; https://www.mhlw.go.jp/stf/seisakunitsuite/bunya/hukushi_kaigo/seikatsuhogo/jisatsu/jisatsuhakusyo2021.html. Accessed 2023.4.1.
6. Nakamoto M, Nakagawa T, Murata M, Okada M. Impacts of Dual-Income Household Rate on Suicide Mortalities in Japan. *Int J Environ Res Public Health.* 2021;18(11):5670.
7. Nakano T, Hasegawa T, Okada M. Analysing the Impacts of Financial Support for Regional Suicide Prevention Programmes on Suicide Mortality Caused by Major Suicide Motives in Japan Using Statistical Government Data. *International Journal of Environmental Research and Public Health.* 2021;18(7):3414.
8. Hasegawa T, Fukuyama, K., Okada, M. Relationships between Expenditure of Regional Governments and Suicide Mortalities Caused by Six Major Motives in Japan. *Int J Environ Res Public Health.* 2022;19:84.
9. Shiratori Y, Tachikawa H, Nemoto K, et al. Network analysis for motives in suicide cases: a cross-sectional study. *Psychiatry Clin Neurosci.* 2014;68(4):299-307.
10. Kawano Y, Matsumoto R, Motomura E, Shiroyama T, Okada M. Bidirectional Causality between Spreading COVID-19 and Individual Mobilisation with Consumption Motives across Prefectural Borders in Japan. *Int J Environ Res Public Health.* 2022;19(15):9070.
11. Linden A. A Comprehensive set of Postestimation Measures to Enrich Interrupted Time-series Analysis. *The Stata Journal: Promoting communications on statistics and Stata.* 2017;17(1):73-88.
12. Linden A. Conducting interrupted time-series analysis for single-and multiple-group comparisons. *The Stata Journal.* 2015;15(2):480-500.
13. Bernal JL, Cummins S, Gasparrini A. Interrupted time series regression for the evaluation of public health interventions: a tutorial. *Int J Epidemiol.* 2017;46(1):348-355.
14. Yoshioka E, Hanley SJB, Sato Y, Saijo Y. Impact of the COVID-19 pandemic on suicide rates in Japan through December 2021: An interrupted time series analysis. *Lancet Reg Health West Pac.* 2022;24:100480.
15. Kim HJ, Fay MP, Feuer EJ, Midthune DN. Permutation tests for joinpoint regression with applications to cancer rates. *Stat Med.* 2000;19(3):335-351.
16. NCI. Joinpoint Regression Program ver4.9.1.0 2022; https://surveillance.cancer.gov/joinpoint/ Accessed 2022.8.1, 2022.
17. Matsumoto R, Motomura E, Okada M. Fluctuation of suicide mortality and temporal causality from unemployment duration to suicide mortality in Japan during 2009-2022. *Asian J Psychiatr.* 2023;84:103574.
18. Okada M. Is an increase in Japan's suicides caused by COVID-19 alone? *Asian J Psychiatr.* 2022;78:103320.
19. Okada M, Matsumoto R, Motomura E, Shiroyama T, Murata M. Exploring characteristics of increased suicide during the COVID-19 pandemic in Japan using provisional governmental data. *Lancet Reg Health West Pac.* 2022;24:100481.
20. Matsumoto R, Kawano Y, Motomura E, Shiroyama T, Okada M. Analyzing the Changing Relationship Between Personal Consumption and Suicide Mortality During COVID-19 Pandemic in Japan, using governmental and personal consumption transaction databases. *Frontiers in Public Health.* 2022;10:982341.
21. MHLW. Patient Survey. 2023; https://www.e-stat.go.jp/en/statistics/00450022. Accessed 2023.1.31, 2023.

© 2023 Okada M et al. *JAMA Network Open.*
