# Supplementary material for: Suicidal Mortality and Motives Among Middle-School, High-School, and University Students
Source: JAMA Netw Open. 2023 Aug 7;6(8):e2328144. doi: 10.1001/jamanetworkopen.2023.28144 (PMC10407687; doi:10.1001/jamanetworkopen.2023.28144)
Supplement: Supplement 2. — Data Sharing Statement [file jamanetwopen-e2328144-s002.pdf]

Data Sharing Statement

Okada. Suicidal Mortality and Motives Among Middle-School, High-School, and University Students. *JAMA Netw Open*. Published August 07, 2023.
doi:10.1001/jamanetworkopen.2023.28144

Data
Data available: Yes
Data types: Other (please specify)
Additional Information: All raw data are publicly available to any persons via Japanese national databases from the Suicide Statistics (SSNPA) collected by the National Police Agency (NPA) and School Basic Survey (SBS) in the Ministry of Education Culture Sports Science and Technology (MEXT).
How to access data: https://www.npa.go.jp/publications/statistics/safetylife/jisatsu.html
When available: With publication

Supporting Documents
Document types: None

Additional Information
Who can access the data: All data has been published by governmental database in Japan.
Types of analyses: All raw data are publicly available to any persons via Japanese national databases
Mechanisms of data availability: All raw data are publicly available to any persons via Japanese national databases
Any additional restrictions: All raw data are publicly available to any persons via Japanese national databases
